# Performances of Conventional and Hybrid Fixed Bed Anaerobic Reactors for the Treatment of Aquaculture Sludge

**DOI:** 10.3390/bioengineering7030063

**Published:** 2020-06-27

**Authors:** Alessandro Chiumenti, Giulio Fait, Sonia Limina, Francesco da Borso

**Affiliations:** Department of Agricultural, Food, Animal and Environmental Sciences (DI4A), University of Udine, via Delle Scienze 206, 33100 Udine, Italy; giulio.fait@uniud.it (G.F.); sonia.limina@uniud.it (S.L.); francesco.daborso@uniud.it (F.d.B.)

**Keywords:** biogas, anaerobic digestion, methane, aquaculture, trout, sludge, wastewater, drum sieve, microfiltration, settling

## Abstract

Aquaculture fish production is experiencing an increasing trend worldwide and determines environmental concerns mainly related to the emission of pollutants. The present work is focused on the improvement of the sustainability of this sector by assessing the anaerobic digestion (AD) of slurry. Wastewater from experimental plants for the production of trout (Udine, Italy) was subject to screening by a drum filter, and then to thickening in a settling tank. The thickened sludge, representing the input of AD, was characterized by total and volatile solids contents of 3969.1–9705.3 and 2916.4–7154.9 mg/L, respectively. The AD was performed in a containerized unit with two digesters (D1 and D2), biogas meters and monitoring of the temperature, pH and redox potential. Both reactors are mixed by a recirculation of the digestate, and reactor D2 is equipped with a fixed bed. The tests were performed at 38 °C with diversified loading rates and hydraulic retention times (HRT). HRT varied from 28.9 to 20.3 days for D1 and from 18.3 to 9.3 days for D2. Methane yields resulted as highest for the hybrid digester with the longest HRT (779.8 NL of CH_4_/kg VS, 18.3 days). The conventional digester presented its best performance, 648.8 NL of CH_4_/kgVS, with an HRT of 20.3 days.

## 1. Introduction

The aquaculture fish production sector is experiencing an increasing trend worldwide, surpassing fishing in 2016 [1]. The diffusion of sea fish farms, mainly characterized by floating cages, and of onshore fresh water intensive facilities, such as tanks, raceways and recirculating aquaculture systems (RAS), positively contributes to fulfill the demand of fish proteins, but conversely determines environmental concerns mainly related to the emission of pollutants. Anaerobic digestion (AD) represents a reliable technology widely implemented in the fields of management of organic wastes, livestock manure and agricultural by-products [2], but it has not been implemented for the treatment of effluents originated from aquaculture. The main advantage presented by the AD process is the production of biogas, a combustible gas formed mainly by CH_4_ and CO_2_, fuel commonly used in combined heat and power (CHP) units for the production of electric and thermal energy, or in some cases, upgraded to biomethane [3]. Furthermore, the AD process provides an important contribution to the protection of the environment by preventing the emission of greenhouse gasses derived from the uncontrolled fermentation of organic matter of different origin, including effluents from farming [2]. The fermentation process determines a sufficient degradation of the organic matter of input feedstocks along with their sanitization, producing an effluent, the digestate, that could be reused as fertilizer as a result of its content of nutrients, with very limited emissions of odors and avoiding spreading fish pathogens [4]. However, the treatment of fish effluents by AD presents some challenges mainly in relation to their excessive dilution, requiring pretreatments for the concentration of the organic matter, to excessive concentrations of free ammonia [5] and relevant concentrations of compounds such as sodium and sulphur, which are present mainly in the case of sea water and can be toxic for methanogenic populations [4].

Kugelman and van Gorder [6] and Mirzoyan et al. [7] treated freshwater aquaculture sludge with a TS content of 4 to 6% in a batch-type digester at 35 °C with hydraulic retention times (HRT) from 30 to 10 days, however, the free ammonia levels found were inhibitory to AD.

Lanari and Franci [5], instead, successfully treated diluted sludge from a trout RAS (1.3–2.4% of total solids (TS), 0.16–0.24 g Tot-N/L) at ambient temperature (25 °C), in a 0.4 m^3^ anaerobic filter filled with polyurethane foam cubes, obtaining biogas production from 49.8 to 144.2 L/day, with a CH_4_ content > 80% and methane yield from 0.40 to 0.46 L/g of volatile solids (VS).

Other scientific papers concerning marine or brackish water effluents or more frequently waste originated from fish slaughtering or industry were recently published, but little is still known about conventional or innovative anaerobic treatment systems for intensive fish farm effluents.

The aim of the present work is to present the results of AD experimental tests conducted on effluents from trout tanks, after filtration and thickening, in a pilot-scale, conventional completely mixed digester and hybrid up-flow, fixed bed digester. In more detail, specific objectives were to study the effects of thickening the influent sludge by sedimentation, the effects sorted by the reduction in HRT and the effects of the increase in the organic load rates (OLRs), both in conventional and hybrid fixed bed reactors.

## 2. Materials and Methods

Tests were performed at the experimental fish production unit of the University of Udine, Italy, where rainbow trout (Oncorhynchus mykiss Walbaum) were bred in 4 circular tanks of 2.1 m^3^, with a fish density changing from 48.9 (initial) to 88.1 kg l.w./m^3^ (final). Wastewater was spilled from the central bottom discharge of each tank and was subject to initial screening by a drum micro-sieve with a 20 µm mesh, and then to thickening in a settling tank.

**Figure 1:**
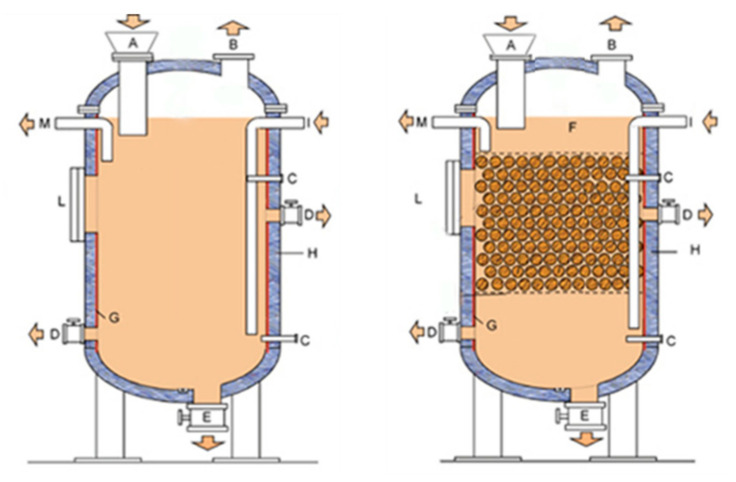
Schematic of the digesters D1 (left) and D2 (right): solid materials input (blocked in the present experimentation) (A), biogas output (B), temperature probe housings (C), sampling points (D), bottom discharge (blocked) (E), fixed bed (F), heating coil (G), insulation (H), sludge input and recirculation ports (I), maintenance openings (L) and digestate output (M).

The anaerobic digestion tests were performed in a containerized experimental unit equipped with a loading tank, with hydraulic mixing, and two anaerobic digesters (Figure 1): the first (D1) is completely mixed by means of programmed recirculation of the digestate, with a useful volume of 280 L; the second reactor (D2) is equipped with a plastic filling media characterized by a relevant specific surface (140 m^2^/m^3^) to promote the development of a fixed layer of anaerobic bacteria, with a useful volume of 260 L. 

The digesters are made of steel, equipped with a loading input located on the top and side overflow discharge; different sampling points are located at different heights, while a valve is installed at the bottom of each digester to allow the removal of eventual sediments. The heating system is represented by a coil of electric resistance externally wrapping the wall of the tanks. The resistances are controlled by Pt100 temperature probes and digital thermostats. The digesters are insulated by a layer of rockwool (40 mm) externally protected by an aluminum sheet. 

Biogas exits the dome of the digesters and flows into a hydraulic valve, that sets the pressure threshold (80 mm H_2_O) and determines the condensation of vapor, and reaches the flowmeters, drum-type (wet-test) Gas Meters (TG1, Ritter DE). Biogas composition is determined by infrared analyzer (Ultramat 23, Siemens, Germany) for (CO_2_ e CH_4_), and by gas suction pump (ProTec, Germany) with H_2_S vials (Kitagawa, Japan).

Process temperature was detected by means of resistive type sensors (Pt100), while pH and Redox potential by electrochemical probes (Steiel, Italy). All the electronic sensors were connected to a PLC (Eukrasia, Italy), which recorded data and also managed all equipment, as loading and mixing pumps.

Input and digestate were weekly sampled to perform the following analyses: Total Solids, Volatile Solids (VS), Chemical Oxygen Demand (COD), Biological Oxygen Deman (BOD_5_), Total Kjeldhal Nitrogen (TKN), P, total alkalinity [8].

The tests were performed in mesophilic conditions (38 °C) with different loading rates intended to test different hydraulic retention times (HRT).

### 2.1. Loading Rates

The tests lasted for a total of 71 days, divided in three periods with different and increasing loading rates (Table 1).

In particular, digester D1 was initially fed with 9.7 L/day of input until day n.52 and from that point on the daily load was increased to 13.8 L/day. Digester D2 was characterized by a higher load than D1 since the beginning, in detail 14.2 L/day up to day n.35, 18.4 L/day from day n.36 to day n.52and 27.3 L/day until the end of the tests. Consequently HRT varied from 28.9 to 20.3 for D1, and was reduced from 18.3 to 14.1 and 9.5 for D2. 

### 2.2. Settling of the Input

The experimentation could be divided in two phases according to the variation in the characteristics of the input due to the introduction of a settling phase performed after the mechanical filtration from day 36. 

The characteristics of the two different inputs will be described in the results.

## 3. Results and Discussion

### 3.1. Chemical Characteristics

Before day 35 (phase F), the effluent was not subjected to settling and hence resulted more diluted (Table 2). Instead, from day 36 to the end of the tests, the effluent presented higher concentrations of all parameters (phase FS, filtration and sedimentation).

Average TS content resulted in 3969.1 mg/L in phase 1 and 9705.3 mg/L in phase 2. The VS/TS ratio remained relatively constant, in the range between 73.4 and 74.1. COD (chemical oxygen demand) and BOD_5_ (biological oxygen demand) respectively resulted in 6433.8 and 3020.0 mg/L in phase 1, and 3020.0 and 6210.0 in phase 2. Alkalinity resulted as quite stable in the two phases, with average values of 1110.2 and 1187.6 mg/L.

On the basis of these results (Table 2) and considering the volumetric loads (Table 1), it was possible to determine the organic load rates (OLR) that characterized the operation of the two digesters during the tests (Table 3). The OLR of reactor D1 was increased from an initial 0.100 to 0.352 kg VS/m^3^ day, while for digester D2, this parameter was increased from 0.158 to 0.750 kg VS/m^3^ day. These results are aligned with the experimentation of Lanari and Franci [5], where an OLR ranging between 0.227 and 0.751 kg VS/m^3^ day was maintained in the AD of wastewater from trout fed at the regime of 1–2% of live weight/day. Alternately, these values can be considered as conservative in comparison with those indicated for swine or cattle manure, which normally are between 1.5 and 6.0 kg/VS day [2], with values below the range resulting in a possible lack of organic matter and low methanogenic bacteria, and values above the risk of acidification as an effect of the accumulation of volatile fatty acids (VFA) [2,3]. Previous tests conducted with the same digesters operating on swine manure presented values of 1.12 and 3.34 kg VS/m^3^ day [2]. This is not to be considered negatively considering that optimal parameters for AD may vary from feedstock to feedstock. 

In terms of COD, the organic load resulted between 0.222 and 0.715 kg COD/m^3^ day for D1, and 0.350 and 1.522 kgCOD/m^3^ day for D2. These values are lower than those maintained by Gebauer [4], who operated with an OLR up to 3.12 kgCOD/m^3^ day on a lab scale with 15 L reactors treating Atlantic salmon sludge from a sieve and air-flushed ribbon strainer.

### 3.2. Characteristics of Digestate and Organic Matter Removal Rate

**Figure 2:**
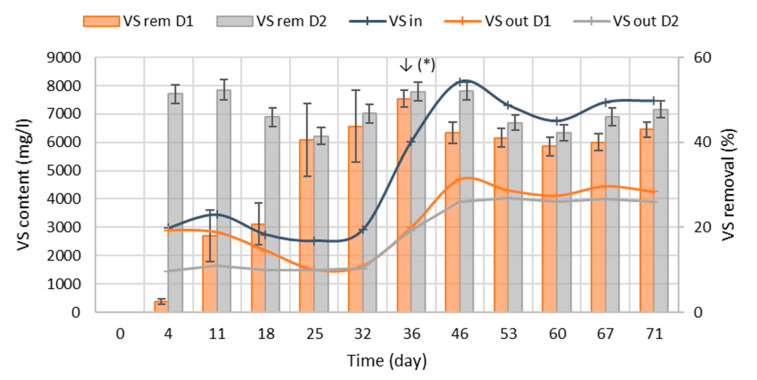
Evolution of VS concentration in the input and in digestates from D1 and D2, and of VS removal rates. ↓(*) = implementation of influent sedimentation.

The concentration of VS in the digestate resulted between 1495.6 and 4691.7 mg/L for D1, and between 1443.1 and 4044.8 mg/L for D2. The VS removal resulted as significant for the anaerobic filter reactor D2 from the beginning of the tests (52.4%) and remained steady during the tests, with an average value of 47.5%; the VS removal rate of D1 presented instead an increasing trend until day 36, reaching a plateau after with an average value of 34.7%, significantly lower than D2 (Figure 2).

The higher performance of anaerobic filters compared with traditional high-rate digesters is well known [2,9], and particularly during the first phase of the process, it can also be partially related to a mechanical retention of solids trapped in the packed bed filter, not only to increased solids degradation. 

### 3.3. Biogas Production

**Figure 3:**
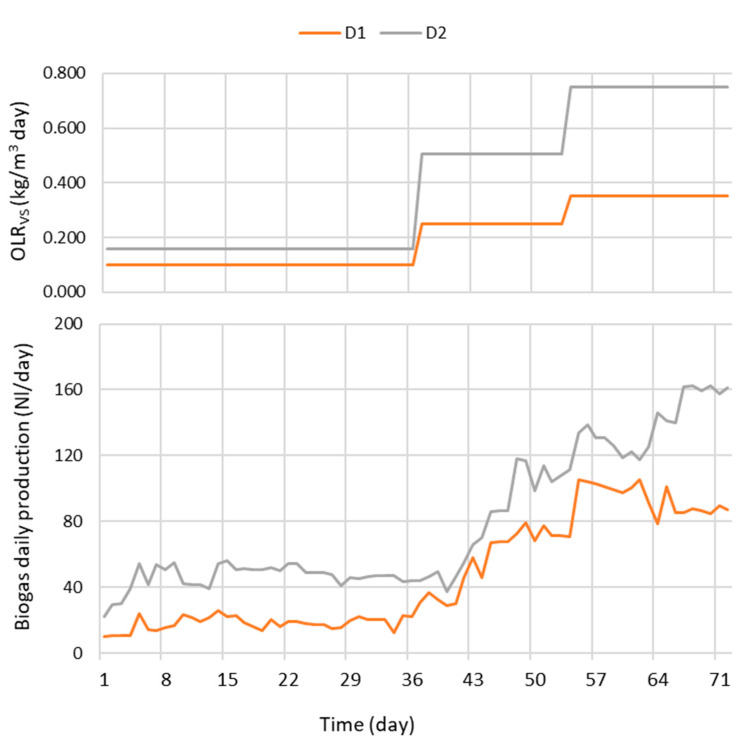
Evolution of OLR_VS_ (above) and of daily biogas production (below).

The evolution of biogas production presented an increasing trend, mainly in relation to the increase in the OLR of the different phases of the tests (Figure 3). 

Digester D2 presented, in general, a higher production than D1. In the first phase of the experimentation, with a higher dilution of the input and HRT (up to day 35), D1 showed a constant production with an average of 30.2 ± 4.7 NL/day, while D2 produced an average of 46.4 ± 7.6 NL/day of biogas. In the following phases, D1 presented at first an increasing trend, then a steady production till the end of the test: the average values resulted in 78.5 ± 28.5 and 139.4 ± 17.2 NL/day in the two periods until days 52 and 71, respectively.

The cumulative production of biogas resulted in 3366.8 and 5650.8 NL for digesters D1 and D2, corresponding to volumetric yields of 0.169 and 0.306 Nm^3^/m^3^day. 

Other studies demonstrate comparable results, with values between 0.130 and 0.377 Nm^3^/m^3^ day, with similar effluents from trout, treated after filtration by the AD process in a pilot-scale system with a fixed bed [5]. Using the same pilot plant, previous tests conducted on filtered swine manure revealed 0.048 to 0.060 Nm^3^/m^3^ day for a traditional digester and 0.111 Nm^3^/m^3^ day for an anaerobic filter [2].

### 3.4. Biogas Quality

**Figure 4:**
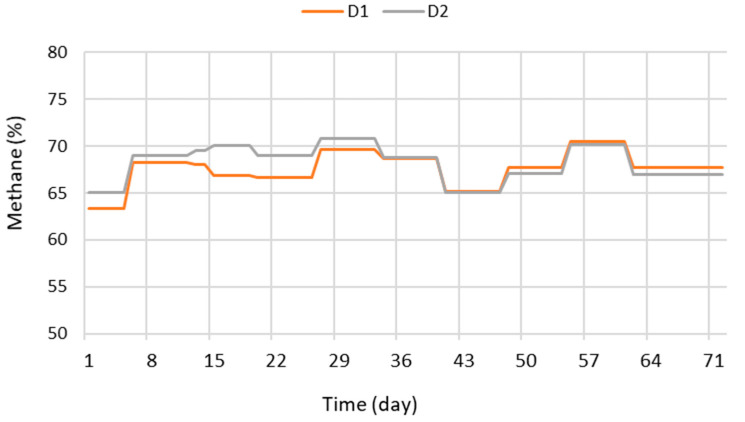
Evolution of methane concentration in biogas.

The concentration of methane in biogas resulted in the ranges 63.3–70.5% and 65.0–70.8% for D1 and D2, respectively (Figure 4), results comparable to those from the AD of swine manure [2] and higher than methane concentrations in biogas from bovine manure [3,10]. The literature on the AD of aquaculture effluents reports variable results in terms of methane concentration in biogas: Lanari and Franci [5], for example, reported 80% of CH_4_ from the AD of trout effluents, while other authors presented values between 48.9 and 57.6% CH_4_ in biogas from Atlantic salmon effluents [4].

### 3.5. Methane Yield

**Figure 5:**
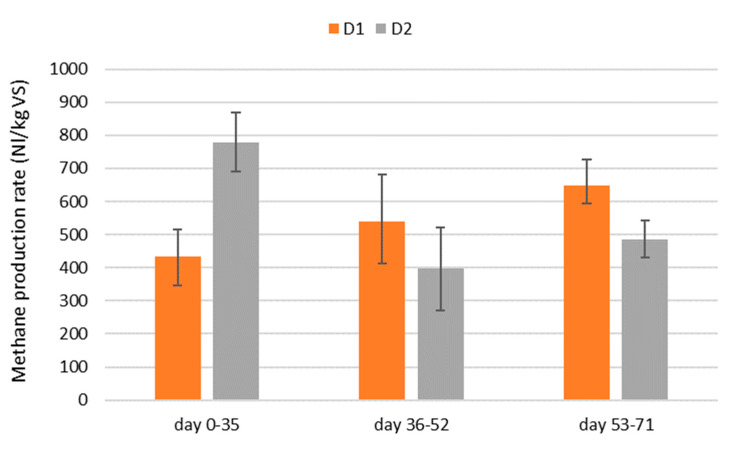
Methane production rates from the 2 digesters during the 3 trial phases.

Methane yield referred to VS resulted very high, between 396.8 (D2, days 36–52) and 779.8 NL/kg VS (D2, days 0–35) (Figure 5). Such values are not common in the treatment of effluents from animal farms or in the agricultural sector.

Comparable methane yields are generally obtained in the case of feedstocks with high energetic content, such as some residues from slaughter houses containing high concentrations of proteins and fat. Pitk and colleagues, for example, reported yields from 390 to 978 m^3^ CH_4_/t in the case of solid slaughterhouse waste rendering products [11]. 

The effluents used in the tests were not characterized in terms of the composition of the organic matter, but the trout feed was characterized by a high lipid content. Furthermore, the highest reported yield from the AD of aquaculture effluents was 460 L/kgVS [5], comparable to the lower values obtained in the current study.

In further detail, D1 demonstrated an increase in yield corresponding to the increase in the OLR, reaching a maximum of 648.8 NL/kgVS with an OLR of 0.352 kgVS/m^3^ day and HRT of 20.3 days. Digester D2 showed a maximum yield of 779.8 NL/kgVS with an OLR of 0.158 kgVS/m^3^ day and HRT of 18.3 days, lower than the HRT of D1. Furthermore, the D2 yield resulted as gradually descending with the increase in the organic load and decrease in HRT, resulting as particularly critical in the period between days 36 and 52, with an OLR of 1.026 kgVS/m^3^ day and HRT of 14.1 days, when a yield of 396.8 NL/kgVS was achieved. Moreover, it must be noted that during the second and third phases, the methane production rates of the two digesters were not so different, as they were during the first phase of experimentation (Figure 5).

## 4. Conclusions

Intensive trout production in tanks determines the production of large volumes of diluted effluents characterized by a low concentration of organic matter.

In the present study, microfiltration and settling were performed to concentrate this wastewater, obtaining an average TS content from 3969.1 (filtration) to 9705.3 mg/L (filtration and settling), with an average concentration of VS ranging between 73.4 and 74.1%TS.

The treated effluents were subjected to the AD process in mesophilic conditions, in pilot-scale conventional and fixed bed hybrid digesters. The evolution of the process was regular and without management problems.

The key results of this experimentation can be summarized as follows:

- The organic matter removal rate resulted as higher in the case of the hybrid digester and with the longest process time (average 52.4% VS removal with an HRT of 18.3 days);
- The CH_4_ concentration in the biogas resulted as relevant and stable during the tests, presenting an average ranging from 63.3% to 70.8%;
- Methane yields resulted as very high, especially for the hybrid digester with the longest process time (779.8 NL of CH_4_/kg VS with an HRT of 18.3 days). The conventional digester presented its best performance, 648.8 NL of CH_4_/kgVS, with an HRT of 20.3 days;
- Operating with the hybrid digester and the lowest process time, corresponding to 9.3 days, it is still possible to achieve an optimal methane yield (486.7 NL CH_4_/kg VS). Applied to a full-scale digester, this solution could determine a reduction in the digestion volume and consequently lower capital and operational costs (i.e., thermal energy for temperature control) while guaranteeing an optimal performance of the process.

## Figures and Tables

**Table 1:** Description of the different loading rates and hydraulic retention times (HRT) for D1 and D2.

	D1		D2	
Days	Load (L/day)	HRT (days)	Load (L/day)	HRT (days)
0–35	9.7	28.9	14.2	18.3
36–52	9.7	28.9	18.4	14.1
53–71	13.8	20.3	27.3	9.5

**Table 2:** Characteristics of the input in the different phases of the tests (average and standard deviation).

	TS (mg/L)	VS (mg/L)	VS/TS (%)	COD (mg/L)	BOD_5_ (mg/L)	TKN (mg/L)	TP (mg/L)	Alkalinity (mg/L)
Phase F Day 1–35	3969.1 ± 209.5	2916.4 ± 341.7	73.4 ± 7.2	6433.8 ± 587.7	3020.0 ± 408.7	205.2 ± 63.4	150.3 ± 21.8	1110.2 ± 71.9
Phase FS Day 36–71	9705.3 ± 1094.0	7154.9 ± 1060.8	74.1 ± 3.3	14511.0 ± 1484.1	6210.0 ± 690	521.3 ± 148.1	333.9 ± 23.9	1187.6 ± 10.8

**Table 3:** Organic loading rates (OLR) in terms of VS and COD maintained in digester D1 and D2 during the experimental trial.

	OLR_VS_ (kg VS/m^3^ day)		OLR_COD_ (kg COD/m^3^ day)	
Days	D1	D2	D1	D2
0–35	0.100	0.158	0.222	0.350
36–52	0.248	0.506	0.502	1.026
53–71	0.352	0.750	0.715	1.522
